# The Collateral Impact of COVID-19 Emergency on Neonatal Intensive Care Units and Family-Centered Care: Challenges and Opportunities

**DOI:** 10.3389/fpsyg.2021.630594

**Published:** 2021-02-24

**Authors:** Loredana Cena, Paolo Biban, Jessica Janos, Manuela Lavelli, Joshua Langfus, Angelina Tsai, Eric A. Youngstrom, Alberto Stefana

**Affiliations:** ^1^Department of Clinical and Experimental Sciences, University of Brescia, Brescia, Italy; ^2^Department of Neonatal and Pediatric Critical Care, Verona University Hospital, Verona, Italy; ^3^Department of Psychology and Neuroscience, University of North Carolina at Chapel Hill, Chapel Hill, NC, United States; ^4^Department of Human Sciences, University of Verona, Verona, Italy

**Keywords:** COVID-19, pre-term infant, neonatal intensive care unit, parents, NICU staff, family-centered care

## Abstract

The ongoing Coronavirus disease 2019 (COVID-19) pandemic is disrupting most specialized healthcare services worldwide, including those for high-risk newborns and their families. Due to the risk of contagion, critically ill infants, relatives and professionals attending neonatal intensive care units (NICUs) are undergoing a profound remodeling of the organization and quality of care. In particular, mitigation strategies adopted to combat the COVID-19 pandemic may hinder the implementation of family-centered care within the NICU. This may put newborns at risk for several adverse effects, e.g., less weight gain, more nosocomial infections, increased length of NICU stay as well as long-term worse cognitive, emotional, and social development. This article aims to contribute to deepening the knowledge on the psychological impact of COVID-19 on parents and NICU staff members based on empirical data from the literature. We also provided evidence-based indications on how to safely empower families and support NICU staff facing such a threatening emergency, while preserving the crucial role of family-centered developmental care practices.

## Introduction

The ongoing pandemic of Coronavirus disease 2019 (COVID-19) has infected, at the time of writing this article, tens of millions of people and contributed to over one and a half million deaths globally (see https://covid19.who.int/). Many governments have imposed regional or national mobility restriction measures in an effort to inhibit its spread. During this global health emergency, special attention has been given to the potential impact of both the severe acute respiratory syndrome coronavirus 2 (SARS-CoV-2) and the measures taken to prevent the virus from spreading to vulnerable populations such as people with serious mental illness (Druss, 2020; Stefana et al., 2020b) and frontline health workers (Chen Q. et al., 2020; Wang J. et al., 2020). However, another vulnerable population, those who are treated, visit or work in the orbit of neonatal intensive care units (NICUs), is receiving less attention than it deserves. Infants who require NICU admission are exposed to a range of intrinsic and environmental factors that can lead, as in the case of pre-term birth, to an increased risk of neurodevelopmental disorders, psychiatric disorders, and chronic disorders involving various organ systems, which can persist from childhood into adulthood or sometimes first manifest in adolescence or adulthood (Saigal and Doyle, 2008; Crump, 2020). However, NICU infants are not the only susceptible population. Indeed, NICU staff members and parents also are vulnerable from a psychological perspective. The NICU staff members frequently encounter work-related stressors that make them prone to burnout and mental health problems (Tawfik et al., 2017; Favrod et al., 2018), whereas parents who have a child being treated in the NICU (i.e., having a critically ill infant and being physically separated from her/him) often perceive this experience as psychologically traumatic (Ionio et al., 2016; Janvier et al., 2016; Sabnis et al., 2019). These issues are likely to be exacerbated by the added burden of the ongoing COVID-19 pandemic, which also hinders the implementation of family-centered care in the NICU, with several negative consequences for the infants. This article aims to provide medical, psychological, and allied health communities with empirical data from the literature on the impact of the COVID-19 pandemic on NICUs' and families. We have also developed evidence-based recommendations for caring family, infant, and NICU staff amid such a challenging pandemic.

## COVID-19 in Fetuses and Newborns

To date, no empirical study has clearly demonstrated the occurrence of intrauterine infection by vertical transmission of SARS-CoV-2 from pregnant women to their fetuses (Karimi-Zarchi et al., 2020; Kimberlin and Stagno, 2020; Schwartz, 2020). However, emerging evidence based on the presence of SARS-CoV-2 specific IgM antibodies in neonates suggests that vertical or peripartum transmission from a woman to her fetus is probable (Shek et al., 2003; Dong L. et al., 2020; Rodrigues et al., 2020; Zeng H. et al., 2020). These results are based on small numbers of cases, thus the proportion of pregnancies affected (which seems to be low; Parazzini et al., 2020) is yet to be determined, and the short- and long-term consequences for babies born to mothers with COVID-19 are still unclear. With regard to COVID-19 post-natal infection in newborns, some studies report cases of neonatal early-onset infection confirmed by nasopharyngeal and anal swabs positive for SARS-CoV-2 assay 36-to-48 h after birth (Wang S. et al., 2020; Zeng L. et al., 2020). Although the majority of infected infants aged <1 year at diagnosis are asymptomatic or have mild-to-moderate symptoms, the prevalence of severe-to-critical symptoms requiring NICU admission is about 10% (Dong Y. et al., 2020). Furthermore, although having COVID-19 during pregnancy may cause some pre-natal problems (Zhu et al., 2020), including pre-term delivery in about one out of four infected pregnant women (Rodrigues et al., 2020), it did not considerably increase the immediate adverse outcome of neonates (Dubey et al., 2020; Parazzini et al., 2020; Rawat et al., 2020; Yee et al., 2020).

## The Psychological Impact of COVID-19

Infections, deaths, and uncertainty about the future as well as the economic and social consequences of essential public health measures used to contain the spread of the virus (i.e., shelter-at-home, quarantine, isolation and lockdown) are playing key roles in the short- and long-term social and psychological impacts of the COVID-19 pandemic (Osofsky et al., 2020; Provenzi and Tronick, 2020). Sheltering in place entails the loss of daily routines and a reduction in social activities and in-person interactions (which, among other things, provide emotional support). In other words, the current pandemic is reducing the quality of individual, family, and social life intrapersonally and interpersonally. Epidemiological studies of the COVID-19 impacts have shown a high burden of psychological distress (anxiety, depression, and stress) among uninfected individuals, particularly among females (Gao et al., 2020; Wang C. et al., 2020). Furthermore, growing evidence indicates that longer duration of externally-imposed social isolation and an inadequate home environment (characterized by small size, low levels of natural luminosity, or limited possibility of privacy) can lead to a wide range of adverse psychological effects, including alienation, diminished self-esteem, helplessness, insomnia, and panic (Brooks et al., 2020; Pancani et al., 2020; Sim et al., 2020), in addition to the aforementioned distress. Moreover, anxiety, anger, and post-traumatic stress disorder can endure for months to years after the end of such mobility restrictions (Brooks et al., 2020).

### Psychological Impact on Parents (and Their Infants)

Though COVID-19 seems to be a less severe illness during pregnancy than previous coronavirus diseases, i.e., Severe Acute Respiratory Syndrome-related coronavirus (SARS) and Middle East Respiratory Syndrome-related coronavirus (MERS), it remains a serious disease as a small number of new mothers may require critical care. There have been few reported cases of both mother and infant deaths in association with COVID-19 (Abou Ghayda et al., 2020; Thornton, 2020), and the factors determining the neonatal mortality seem to be a consequence of pre-term birth rather than of infection with SARS-CoV-2 (Hessami et al., 2020). Pregnant women fear that they may be infected and transmit the harmful infection to their baby, damaging or causing him/her physical pain, whereas fathers are primarily (but probably not exclusively; Stefana and Lavelli, 2018) worried about the risks for both their partners and their babies.

Given the current coronavirus-related restrictions, fathers often are kept out of the delivery room and/or the obstetrics and gynecology ward during delivery in an effort to protect patients and staff from infection (Carroll et al., 2020; Gressier et al., 2020). Mothers with suspected, probable, or confirmed COVID-19 who must take care of their infants by themselves (due to their partners being kept from the ward) must apply standard precautions (e.g., hand hygiene before and after contact with the baby, use a medical mask when near the baby, and routine disinfection of surfaces and objects used) to preserve physical health. Such precautions could be psychologically demanding and complicate the mother's relationship with her baby. The first contacts between mother and newborn are crucial to start the bonding process (Johnson, 2013; Widström et al., 2019). Immediate skin-to-skin contact and breastfeeding within 2 h following delivery make new mothers more sensitive to the infant's needs, and the infant's innate interest toward social stimuli meets a constellation of species-specific caregiving bonding-related behaviors such as looking, vocalizing, positive facial affect and affectionate touch that appear soon after birth (Feldman and Eidelman, 2007; O'Higgins et al., 2013). In contrast, post-natal separation disrupts the establishment of the early parent-infant physiological/emotional connection (Flacking et al., 2012; Welch and Ludwig, 2017) and leads to inadequate mother-infant relationships that can result in long-term negative consequences for the child's cognitive, socio-emotional, and physical development, as well as interpersonal relationships (Johnson, 2013). Adhering to the coronavirus-related restrictions, despite the World Health Organization recommendation that “mothers with suspected or confirmed COVID-19 should not be separated from their infants” (WHO, 2020), means that mothers who are suspected or confirmed to have an infection but are generally in good health are not allowed to care for and feed for their babies according to standard guidelines in some countries and hospitals (WHO, 2002), even when applying necessary precautions for infection prevention and control (Davanzo et al., 2020; Stuebe, 2020). Furthermore, in some countries, these mothers are not allowed skin-to-skin contact in the delivery room or in the ward (this is a pivotal aspect because the early experience of skin-to-skin contact can lead to decreased nosocomial infections and pain perception and to improved breastfeeding, sleeping patterns and neurodevelopmental outcomes; Holditch-Davis et al., 2014; Lumbanraja, 2016; Johnston et al., 2017; Casper et al., 2018; Karimi et al., 2019). These restrictions adversely impact mothers' mood, self-esteem, self-confidence, and confidence in their abilities to care for their infant (Morelius et al., 2005; Bigelow et al., 2014; Krol and Grossmann, 2018; Pineda et al., 2018).

This situation is even more complex and critical in the case of high-risk infants. Even in a non-pandemic period, having a child admitted to a NICU is a traumatic and stressful experience for most parents (Stefana and Lavelli, 2016; Sabnis et al., 2019), mainly because of the unfamiliarity and intimidating intensive care unit environment, the limited ability to provide care for their child, and the uncertainties and worries about their child's outcomes (Obeidat et al., 2009; Stefana et al., 2018). Parents of infants hospitalized in a NICU are at high risk for developing anxiety and depressive symptoms or disorders (Mendelson et al., 2017). They need and desire comprehensive, timely, and clear information about their baby as well as emotional support (Franck and Spencer, 2003). Furthermore, these parents are likely to develop high levels of stress and feelings of guilt and shame, e.g., for not being able to provide care for their hospitalized child in the way they want to or from the sense that they are responsible for their infant's pre-term birth (Flacking et al., 2007; Roque et al., 2017; Stefana et al., under review). During the ongoing pandemic, infants are admitted in an isolated room of the NICU, and mothers with suspected or confirmed COVID-19 sometimes may be totally separated from their child for days or even weeks. In cases where the other parent is also infected, they cannot visit the infant until the test results return negative. Furthermore, in an effort to reduce the risk of SARS-CoV-2 transmission, many NICUs have reduced parental (especially paternal) and family visitation privileges (Cavicchiolo et al., 2020a; Murray and Swanson, 2020) regardless of the other parent's chance of being infected. Despite parents' understanding of the need for visitation restrictions, they are seriously concerned about their ability to visit, care for, and bond with their hospitalized infants (Muniraman et al., 2020).

Forcing a parent to be separated from their newborn child is a devastating experience that adds much to the distress of NICU admission (Bembich et al., 2020), and could negatively impact child development and family well-being in the long term (Erdei and Liu, 2020). Adverse consequences include reduced opportunities for breastfeeding and skin-to-skin touch and holding (Furlow, 2020), delayed and reduced parent–infant interactions (which play a crucial role in early regulation of the stress response and provide the foundations for the development of mutual regulation; Stefana and Lavelli, 2017; Stefana et al., 2020a; Lavelli et al., under review), reduced maternal bonding and infant attachment, parental emotional issues (Franck and Spencer, 2003; Latva et al., 2004; Mäkelä et al., 2018), later parental mental well-being (Lean et al., 2018), and worse infant/child developmental outcomes (Turpin et al., 2019; Cheong et al., 2020). For these reasons, the United States Centers for Disease Control and Prevention (2020) suggest that “the risks and benefits of temporary separation should be discussed by the healthcare team.”

Likely, the adverse effects experienced by parents following their infant's admission to the NICU are more severe and long-lasting during the COVID-19 crisis because many traumatic experiences could have a cumulative effect (Khan, 1963; Sacchi et al., 2020). A recent systematic review and meta-analysis, aimed to estimate the effect of the COVID-19 pandemic on both pregnant and post-partum women's mental health, found that pregnant women and new mothers of full-term and healthy infants report substantially higher levels of anxiety and depression symptoms compared to similar pre-pandemic cohorts (Yan et al., 2020). More specifically, the authors found that the prevalence rates of anxiety and depression among pregnant women during the pandemic were, respectively 37 and 31%, whereas the prevalence of post-partum depression was 22% (the pooled prevalence rate of post-partum anxiety was not evaluated due to the limited data available). Before the COVID-19 pandemic, the estimated prevalence of anxiety symptoms among pregnant women was between 18 and 25% (Dennis et al., 2017; Cena et al., 2020a), while the pooled prevalence of depression among new mothers was between 18 and 20% (Woody et al., 2017; Cena et al., 2021). Furthermore, the levels of anxiety and depression of parents of children who are not infected but are hospitalized during the COVID-19 pandemic are more serious than that of parents of children hospitalized during non-pandemic periods (Yuan et al., 2020). Under such distress, previous evidence suggests that some of these parents may also develop post-traumatic stress disorder (Ursano et al., 2009; Cukor et al., 2011). Finally, reduced maternal and paternal mental health may also lead to additional risk factors for child neurodevelopmental disorders (Giallo et al., 2014; Cena et al., 2020b). For example, two recent systematic reviews found that maternal pre-natal stress is associated with an increased risk of poor socio-emotional development (e.g., difficult temperament, behavioral dysregulation; Madigan et al., 2018) as well as of autism spectrum disorder and attention-deficit hyperactivity disorder in the offspring (Manzari et al., 2019). Furthermore, a longitudinal study involving 3,741 father-child dyads found that fathers' high post-natal distress and low parenting self-efficacy were associated with lower parenting consistency and higher levels of hostile parenting when offspring were aged 4–5 years, and poorer child emotional-behavioral outcomes at 8–9 years (Rominov et al., 2016).

### Psychological Impact on NICU Staff Members

NICU staff members are the key players in the provision of infant health care and family-centered care. Efforts to maintain high-quality care can be emotionally demanding, due to factors such as frequent changes in technology and guidelines as well as recurrent occupational exposure to the pain and distress of high-risk neonates and their families; this can negatively impact both personal and professional well-being and performance (Van Mol et al., 2015; Weintraub et al., 2016; Tawfik et al., 2017). Thus it is not surprising that even in normal conditions, burnout (defined as a state of fatigue, detachment, and cynicism) affects 25–50% of NICU professionals (Profit et al., 2014; Tawfik et al., 2017; Barr, 2020).

The ongoing global health emergency is a stressful situation for NICU staff both personally as people and professionally as clinicians. Pandemic-related factors such as (i) over-work or work with long shifts, (ii) wearing additional personal protective equipment, which has been described as necessary but time-consuming and disruptive to clear communication with parents (Semaan et al., 2020; Cena et al., under review), (iii) being unable to act according to their own values, the values of the patient's family, or the values of the family-centered care model (i.e., because of pandemic-related policies enforcing social distancing and other measures that are not typical in NICU patient care), (iv) difficulties in meeting the emotional needs of hospitalized infants and their families while also safeguarding their own health, (v) anxiety and fear about their personal physical safety (Chang et al., 2020) as well as that of friends and family members (whom they could infect while asymptomatic), (vi) emotional pain for the loss of infected friends/relatives/colleagues, and (vii) restrictions on personal and social activities are contributing to increased psychological stress in these people. It follows that these professionals are at higher than average risk for burnout (Profit et al., 2014; Crowe et al., 2020), a condition that poses additional challenges for family-centered care. Thus, NICU staff members are in a continuously stressful situation both in the workplace and in their personal lives.

## Supporting Persons and Strengthening NICUs

Next we offer suggestions on how to support and empower both NICU parents and staff, and strengthen NICU systems, while emphasizing the role of Family-Centered Care in the NICU during the COVID-19.

### Family-Centered Care

Family-centered care in the NICU requires as primary components the family's presence in the ward, family support, communication with family members, use of specific consultations and NICU team members, and operational and environmental issues (Davidson et al., 2017). Despite the fact that family-centered care is challenged by the current COVID-19 pandemic, leading to visitation restrictions and indications for physical distancing, its goals must remain the same, though adapted to and focused on maintaining family integrity and respecting the role of family members as care partners with whom to collaborate (Papadimos et al., 2018; Hart et al., 2020). Given that several important practices in typical family-centered care may not be feasible in times of pandemic, family-centered care in the NICU must undergo specific adaptations in order to be accomplished in the midst of the COVID-19 pandemic (see Table 1).

**Table 1:** NICU family-centered care in pre-COVID-19 and COVID-19 pandemic.

**NICU family-centered care concept**	**Pre-COVID-19 pandemic**	**COVID-19 pandemic**
**Communication**		
Face-to-face, in-person communication	+	–
Structured communication	+	–
Telephone calls	+	+
Video calls	+	+
**Family support**		
Peer-to-peer support	+	(–)
Family education programs (e.g., leaflets, videos)	+	+
Patient-diaries by NICU-staff	+	–
Family-authored diaries	+	+
**Family presence**		
Open or flexible presence at the bedside	+	–
Participating in team rounds	+	–
**Special consultations**		
Clinical psychologists or psychotherapists	+	(–)
Family care specialists	+	(–)
Family navigators (e.g., care coordinator or communication facilitator)	+	(–)
Spiritual advisor or chaplain's support	+	(–)

*Table adapted from Zante et al. (2020)*.

*+ concept widely applicable, – concept challenging to apply, (–) concept that could be technically adapted (e.g., through telephone or video calls)*.

### Supporting Parents

When parents' visitation is limited or denied, communication between them and the NICU team should include a video component. Real-time videoconferencing is a means for parents to communicate (and collaborate) with NICU staff and to see their infant (Lindberg et al., 2009; Gund et al., 2013; Epstein et al., 2015; Joshi et al., 2016). It is essential in the current health emergency that parents can see their baby via video when they cannot be or stay with them in the NICU (Epstein et al., 2017), as viewing their newborn on a camera reduces parental stress and anxiety (Rhoads et al., 2015a,b).

A further consequence of visitation restrictions and rules for social distancing is the loss of in-person, peer-to-peer support for NICU parents (Hall et al., 2015). Support groups have a beneficial, normalizing effect on the parental role, emotions, control, trust, coping, and adaptation to parenthood reality (Dahan et al., 2020). More generally, offering peer support is recommended as an integral and crucial component of family-centered care and comprehensive family support (Hall et al., 2015, 2016). Although meeting in-person appears to be preferable, both individual and group peer support interventions offered by telephone or via the internet appear to be beneficial (Hall et al., 2015) when the communications are managed by the same staff who would normally deliver that information personally inside the NICU (i.e., when not managed by a “stranger”). Thus, the best solution in the time of COVID-19 seems to be providing peer support by video and voice calls. NICUs should develop or implement internet-based peer support programs, and offer a comprehensive training program to both veteran parents (i.e., parents who have had previous experience with their own infant in a NICU, have participated in the family-integrated care program, and now provide peer-to-peer support) and NICU staff members who facilitate the support.

In order to ensure that support by mental health professionals continues to function, perinatal psychiatric and psychological services should be implemented through telepsychiatry (see www.psychiatry.org/psychiatrists/practice/telepsychiatry) and telepsychology (see www.apa.org/practice/guidelines/telepsychology and https://w.wiki/NYz) (Hermann et al., 2020; Perrin et al., 2020; Zork et al., 2020). This might include the development of telephone helplines manned by mental health specialists, specifically addressing the needs of parents with hospitalized infants.

Finally, it is crucial that when parents are allowed to visit in the NICU, the healthcare team put in place all the interdisciplinary recommendations for educational and emotional support (Hynan et al., 2015) and for encouraging and involving them in the care of their baby (Craig et al., 2015). Regarding emotional support, the implementation of evidence-based assessment (EBA) and treatment appear to be essential to reduce parents' burden at individual and public health levels. An EBA model that could be usefully adopted by NICU mental health professionals is that devised by Youngstrom and colleagues (Youngstrom, 2013, 2014; Youngstrom et al., 2015, 2017, 2018; Youngstrom and Van Meter, 2016, 2018; Youngstrom and Prinstein, 2020). This EBA 2.0 model (see Table 2) combines empirical research and pragmatism of application to identify the most appropriate measurements and sequence their order to minimize redundancy and unnecessary testing. Such an effective and efficient assessment process is crucial because it leads to more accurate diagnosis, appropriate intervention, better treatment matching, and enhanced outcomes.

**Table 2:** Strategies for adding Evidence-Based Assessment techniques for mental health issues to the NICU (adapted from Youngstrom et al., 2017).

**Assessment step**	**Suggestions for doing in NICU**
**Preparatory work before seeing patient**	
A. Plan for most common issues	Have screening tools and tip sheets for anxiety, depression, acute and post traumatic stress disorders (both parent- and staff-facing); burnout
B. Benchmark base rates for issues	Benchmark local rates against prior years, regional and national data, and/or published estimates
**Admission (“Prediction phase”)**	
C. Evaluate risk and protective factors	Make short checklist of key risk, protective factors to improve consistency and coverage
D. Revise probabilities based on intake assessments	Have cheat sheet with updated probabilities based on screening results and suggested language for follow-up (Well-supported staff could use free online calculators, nomograms, more traditional Evidence-Based Medicine.)
E. Gather collateral, cross-informant perspectives	Assess both parents and relatives (e.g., grandparents) when possible, and share psychoeducational resources (infographics, tip sheets, online tools). Regarding staff members, information should be collected also from NICU colleagues and managed by mental health professional not affiliated with the NICU.
**Targeted follow-up (“Prescription phase”)**	
F. Add focused, incremental assessments	If using ultra-brief screeners, have full-length assessments ready for follow-up. Family can do quickly while on unit, or from home. Often same tool can used as Patient Reported Outcome (PRO).
G. Brief structured interviews	Have short, structured interviews for common mental health issues (e.g., PRIME-MD, DIAMOND) and orient staff to using anxiety, mood, trauma modules.
H. Case re-formulation and goal-setting	If findings suggest mental health issue, provide referral options, psychoeducational resources.
X. Learn and use client preferences	Discuss options and risks and benefits; address common concerns or misconceptions, problem solve around barriers
**Monitoring throughout the infant's stay in the NICU and after discharge (“Process Phase”)**	
I. Goal setting: Milestones and outcomes	Have “cheat sheet” with benchmarks for Minimally Important Difference (MID), clinically significant worsening or improvement on PRO (Step F)
J. Progress tracking	Can repeat PRO (Step F) weekly while in the NICU and at each follow-up visit after infant's discharge.
K. Maintaining gains	Celebrates gains; and plan for continuity of care and ongoing support for family. Develop list of key indicators, recommendations about next action if starting to worsen.

*CC BY 4.0 Eric Youngstrom, PhD*.

*See https://en.wikiversity.org/wiki/Evidence-based_assessment/NICU for links to tools*.

### Supporting and Empowering NICU Staffs

From the above, it follows that it is vital to adequately support NICU staff members in maintaining their security and safety (e.g., personal protective equipment to protect themselves) and, more generally, that hospital institutions make them feel cared for. At the same time, being on the front line to cure and care for the most vulnerable (especially newborns and their families) and being a member of a highly specialized team who bravely faces this threat while continuing to do their jobs are elements that can encourage and even make healthcare providers fittingly proud of themselves and their efforts (Barello et al., 2020a,b). In addition to the above responsibilities, usually NICU staff are also the primary point of contact with the general health system for the parents of hospitalized infants, and as such could (and should) also be the first observer/responder for both SARS-CoV-2 and mental health conditions for many of these parents. Thus, NICU staff need specific training to recognize the signs and symptoms of both COVID-19 and the most common post-partum mental disorders (i.e., anxiety, depression, and psychological distress). Additionally, in this scenario, NICU staff would ideally have (i) accurate and clear guidelines, (ii) access to online screening tools to explore their own mental health status and determine whether they should contact a mental health professional (e.g., www.hgaps.org/assessment-center.html and www.dbsalliance.org/education/mental-health-screening-center/), (iii) the provision of a dedicated psychological help service for healthcare professionals located in the hospital or through a telephone helpline staffed by mental health professionals not affiliated with the NICU, (iv) training on COVID-19 management, (v) training on the use of mobile and web technologies needed to provide support at a distance to parents, (vi) training and access to brief anxiety and depression scales to use for monitoring families' distress (e.g., www.hgaps.org/assessment-center.html and www.dbsalliance.org/education/mental-health-screening-center/), and (vii) a forum for discussion, advice and support from colleagues. These support and training measures are essential and must be developed and implemented, particularly if the pandemic and its aftermath will continue for a long time. If professionals do not have accessible resources and support to take care of their physical and mental health, they will not be able to deliver the appropriate care and critical services to the most vulnerable populations, including infants and NICU families, during this pandemic.

### Strengthening NICU Systems

The COVID-19 global health crisis is a disaster; however, it can also be an opportunity (Stefana et al., 2020c; Youngstrom et al., 2020) to improve health care systems and services by including an increased number of NICU staff members, adequate resources and training, and improved visitation policies for family members of hospitalized infants. Before the COVID-19 pandemic, there was a widespread and substantial shortage of NICU medical and nursing staff (Rogowski et al., 2013; Gagliardi et al., 2016; Bliss, 2017) the current crisis has highlighted. Indeed, three of ten NICUs were already understaffed compared to national guidelines (Rogowski et al., 2013). This is particularly important in pandemic times because understaffing is associated with children's adverse outcomes, including a heightened risk of nosocomial infection on very-low-birth-weight infants (Rogowski et al., 2013). Despite understaffing being a significant risk factor for poor patient outcomes, it is infrequently addressed by interventions (Stapleton et al., 2016).

In many cases, the needed restrictions and containment measures (Cavicchiolo et al., 2020a,b; De Rose et al., 2020) that are in place to deal with the COVID-19 emergency are exacerbating the problems associated with meager adoption of family-centered care principles in NICUs. During these months, it has been common to hear colleagues say that “COVID has made us go back decades in the quality of family support we provide.” This is a serious negative development because care should be all the more humane and person-centered during the COVID-19 pandemic; a goal that becomes fully achievable only through a strengthened involvement of patients' families (Coulter and Richards, 2020) and the support and empowerment of frontline healthcare workers. The development of online support groups, video and messaging platforms to increase communication between families and providers, as well as peer support, all are innovations that should continue even after the pandemic ends. As such, the present healthcare crisis can increase the awareness of healthcare specialists about the critical need to enable open access of families to the intensive care unit environment, and an active engagement of parents in the primary care of hospitalized newborns and infants at risk. The tools and techniques developed in response to the disruption of the system can ratchet practice forward.

## Conclusions

The COVID-19 pandemic has dramatically changed the lifestyle of people worldwide, while disrupting healthcare services and systems, including NICUs. The mitigation strategies adopted to manage the pandemic have upset care delivery for high-risk newborns and their families, and the mental health legacy of the pandemic will likely endure – for both NICU staff and family members – long after the acute phase (Erdei and Liu, 2020; Lemmon et al., 2020). It is vital to deepen the understanding of how the pandemic has influenced family-centered care practices and dynamics in NICUs, gauging the psychological impact of COVID-19 on parents and frontline professionals. This article provides evidence-based strategies to aid NICU staff members engaged in ensuring high-quality care and supporting critically ill newborns and their families (Tscherning et al., 2020). We proposed several ways to safely support and empower NICU staff and enhance family-centered developmental care practices, without increasing the risk of contagion. Apart from evidence-based training on cutting-edge COVID-19 management tools, high priority should be given to the preservation of family-centered care principles, including parents' presence in the NICU, parent-infant physical and emotional closeness, and parental involvement in the infant's care. Furthermore, NICU systems should implement evidence-based assessment and treatment for parental distress while providing peer support for parents by video and voice calls. Finally, NICU systems should ensure dedicated psychological help services for healthcare professionals, being particularly exposed to a higher risk of burnout COVID-19 related.

## Author Contributions

AS designed the study. AS, EAY, PB, JJ, ML, JL, AT, and LC contributed to the manuscript writing. All authors contributed to the article and approved the submitted version.

## Conflict of Interest

The authors declare that the research was conducted in the absence of any commercial or financial relationships that could be construed as a potential conflict of interest.
